# Ultrasound-Induced Drug Release from Stimuli-Responsive Hydrogels

**DOI:** 10.3390/gels8090554

**Published:** 2022-09-01

**Authors:** Tyus J. Yeingst, Julien H. Arrizabalaga, Daniel J. Hayes

**Affiliations:** 1 Department of Biomedical Engineering, The Pennsylvania State University, University Park, Centre County, PA 16802, USA; 2 Materials Research Institute, Millennium Science Complex, The Pennsylvania State University, University Park, Centre County, PA 16802, USA; 3 The Huck Institute of the Life Sciences, Millennium Science Complex, The Pennsylvania State University, University Park, Centre County, PA 16802, USA

**Keywords:** hydrogels, polymers, stimuli-responsive, ultrasound, smart hydrogels, drug delivery, Tissue engineering, cancer therapy, controlled drug release, thermoresponsive materials

## Abstract

Stimuli-responsive hydrogel drug delivery systems are designed to release a payload when prompted by an external stimulus. These platforms have become prominent in the field of drug delivery due to their ability to provide spatial and temporal control for drug release. Among the different external triggers that have been used, ultrasound possesses several advantages: it is non-invasive, has deep tissue penetration, and can safely transmit acoustic energy to a localized area. This review summarizes the current state of understanding about ultrasound-responsive hydrogels used for drug delivery. The mechanisms of inducing payload release and activation using ultrasound are examined, along with the latest innovative formulations and hydrogel design strategies. We also report on the most recent applications leveraging ultrasound activation for both cancer treatment and tissue engineering. Finally, the future perspectives offered by ultrasound-sensitive hydrogels are discussed.

## 1. Introduction

Stimuli-responsive drug delivery systems enable the delivery of payloads on-demand, at a specific time, and at a specific location [1,2,3,4]. These platforms can be designed to respond to a variety of different stimuli, either internal such as redox, pH, or enzymes, or external physical triggers such as magnetic field, ultrasound, light, electricity, or temperature [5,6,7,8,9,10].

For the past 70 years, ultrasound has been extensively used as a diagnostic tool [11,12]. However, it has recently been applied to a broad range of therapeutic applications such as the treatment of vascular thrombosis by dissolving clots, the ablation of tumors, and the healing of bone fractures [12,13,14]. Ultrasound has proven to be both safe and ethical for in vivo use in a variety of applications [15,16]. Ultrasound also induces biological effects that are beneficial for therapeutic applications. It enhances transdermal drug delivery, enhances uptake in cells and tissues, and facilitates wound healing [13,17,18,19,20,21]. Ultrasound provides the capability for a wide variety of applications in the biomedical field including imaging [22], clinical diagnosis [23], therapeutics delivery [20,24,25], detection [26], sensing [27,28], the initiation of chemical and biological processes [29,30,31], and the release of signaling molecules [32].

Ultrasound also possesses several advantages as a stimulus for drug delivery platforms. It allows for the control of material properties and functions both easily and safely. It is non-ionizing, non-invasive, localized, and allows for deep tissue penetration and spatiotemporal control [33,34,35,36]. Ultrasound possesses the ability to be focused and localized to a small region of interest [15,37]. Acoustic energy can then be transferred by high or low intensity focused ultrasound either via thermal or non-thermal mechanisms [24]. A wide variety of polymeric carriers have been developed for ultrasound-responsive drug delivery. The possibilities offered by micelles, nanobubbles, nanodroplets, emulsions, and vesicles have already been thoroughly reviewed [38,39,40,41,42,43,44,45]. Consequently, we will only focus on the prospects offered by hydrogels as ultrasound-responsive delivery platforms. The aim of the present article is to highlight the mechanisms of inducing payload release via ultrasound, examine the latest innovative strategies employed to rationally design hydrogels, and describe their successful applications.

## 2. Acoustics

The developing field of responsive hydrogels is reaching new intersection points with external stimulus triggers. Recent developments have brought stimuli-responsive hydrogels into the field of acoustics and ultrasound. In this case, the acoustics field can be defined as the use of mechanical waves for energetic transfer in materials such as solids, liquids, or gases [37,46]. The transfer of energy into and through materials is then converted into specific acoustic responses for each hydrogel. These acoustic responses include payload delivery, modulation of material properties, initiation of biochemical processes, directed assembly, actuation, locomotion, or sensing [37,47,48,49,50].

The positive characteristics of ultrasound acoustics are frequency, wavelength, time, and transmission loss [51]. While acoustic frequencies range anywhere from 1 Hz to over 100 GHz, ultrasound frequencies only make up the range of 20 kHz to 50 MHz [37,46]. This range of frequencies is particularly interesting since it is outside of the range of human hearing [37]. Additionally, these ultrasound frequencies have generally small wavelengths in water, making them extremely compatible with responsive systems used within the human body [52]. The short time scales of ultrasound frequencies also make them extremely efficient in energy exchange [53]. Another positive characteristic is the low amount of transmission loss within the human body in this frequency range [37]. Due to these positive characteristics, ultrasound is an ideal external trigger for stimuli-responsive hydrogels.

## 3. Acoustic Mechanisms

**Figure 1:**
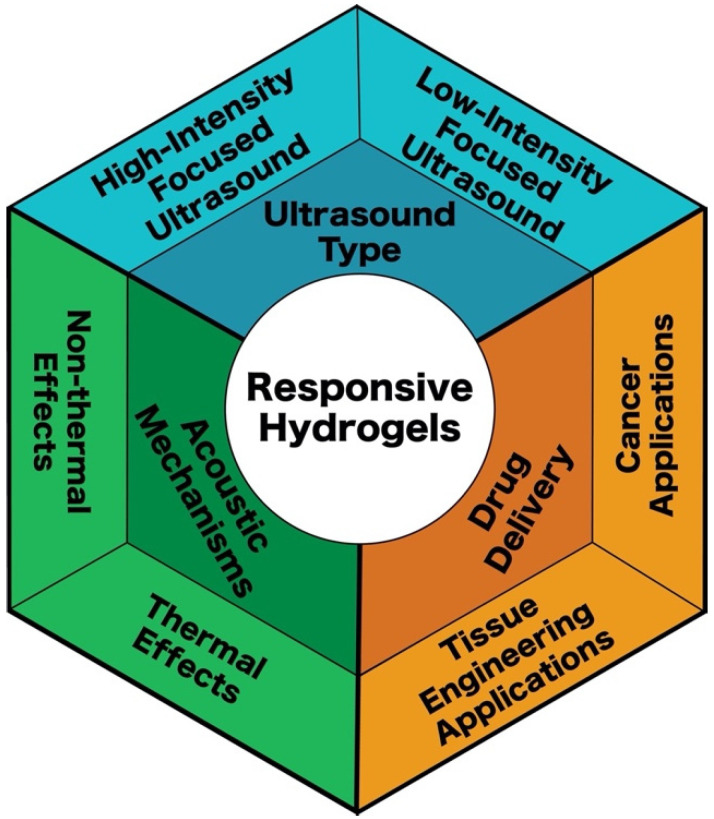
Ultrasound-sensitive hydrogels are designed to respond to ultrasound (either low or high-intensity) via thermal or non-thermal effects. Applications for these drug delivery systems include cancer therapy and tissue engineering.

When using ultrasound acoustics on stimuli-responsive hydrogels, acoustic mechanisms are the pathway in which energy is transferred to induce a response. Acoustic responses typically involve work that is not directly correlated to acoustic waves. The acoustic waves are instead used for energetic transfer through both thermal and non-thermal mechanisms within a responsive hydrogel (Figure 1).

**Figure 2:**
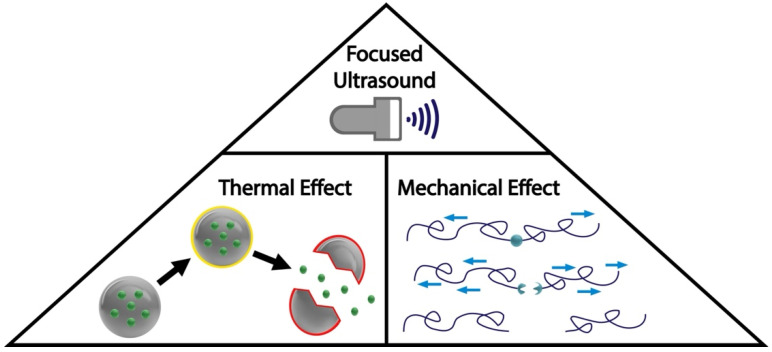
Mechanisms of the ultrasound response of hydrogels. Acoustic energy can be transferred either via thermal or mechanical effects.

The thermal mechanism (Figure 2) is the pathway in which acoustic energy is transferred into thermal energy. The increase in temperature caused by ultrasound irradiation enhances drug diffusion and increases cell permeability [54]. Positive results have been observed with ultrasound-triggered drug release in thermosensitive hydrogels containing colloids such as nanoparticles [55], liposomes [56], and micelles [57]. While the power of high-intensity focused ultrasound is proven to be useful for drug delivery, damage to surrounding cells should be accounted for when considering long-term hyperthermia [33,58,59].

The non-thermal mechanism (Figure 2) is the pathway in which acoustic energy is transferred into mechanical energy in the form of oscillation and force [33]. This mechanical energy can take the form of acoustic cavitation. Cavitation is the formation of bubbles within a material, in which the bubble rapidly oscillates and then collapses within itself [60]. Cavitation has been used for drug delivery for chemotherapy [61] and bone regeneration [62,63]. Mechanical energy can also take the form of ultrasonic mechanical force. This mechanical force can be used to cleave unstable bonds [33]. Acoustic radiation force is another form of mechanical energy derived from ultrasound. The forces created by the acoustic waves act on the particles suspended within a fluid, these particles then move, cluster, and interact with one another [64]. The movement and interaction of these particles create acoustic radiation forces, which when paired with low-intensity focused ultrasound can be used for drug delivery and bone regeneration [65,66].

High-intensity focused ultrasound and low-intensity focused ultrasound prove to be effective in drug delivery using both thermal and non-thermal mechanisms in stimuli-responsive hydrogels. High-intensity focused ultrasound is extremely effective when inducing drug release, however possible damages and challenges may occur for sensitive biological systems [37,67]. While low-intensity focused ultrasound may be less powerful, it is at lower risk of damaging sensitive biological systems [68,69]. In scenarios using thermo-responsive hydrogels with hyperthermia as the thermal mechanism, high-intensity focused ultrasound would be ideal [33]. While both forms of focused ultrasound have respective challenges, it is seen that each can be useful for different applications.

Thermo-responsive and ultrasound-responsive hydrogels respond positively to ultrasound acoustics, making focused ultrasound an excellent external trigger for both systems. Both types of hydrogels prove to be responsive to ultrasound stimulation due to the combination of hyperthermia and sonoporation induced by focused ultrasound [33,58,70]. While different mechanisms exist for both types of hydrogels, each transfers acoustic energy into a form of work proven to be useful for drug delivery. Specifically, drug delivery for the purpose of cancer therapeutics and tissue engineering. Thermo-responsive materials paired with focused ultrasound have been used for both cancer treatments [42,71] and tissue repair [72]. Ultrasound-responsive materials paired with focused ultrasound have been used for both chemotherapy [73] and bone tissue engineering [63,74].

## 4. Designing Hydrogels for Drug Delivery

**Figure 3:**
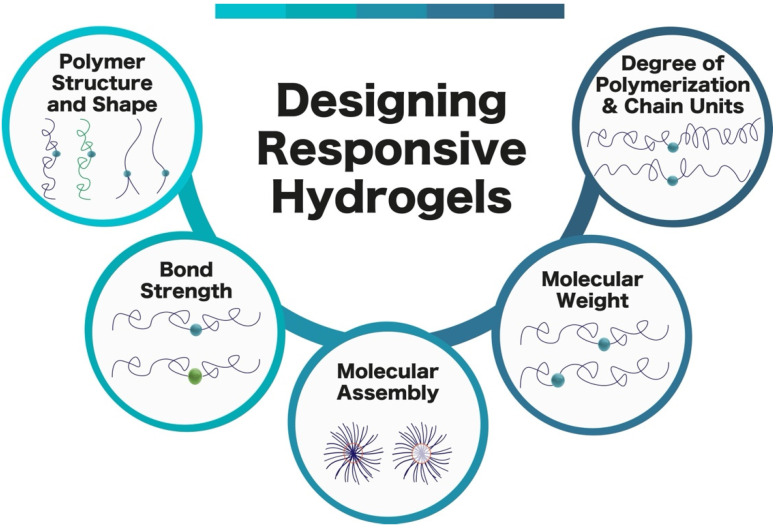
Overview of parameters influencing the design of ultrasound-responsive polymer-based hydrogels.

Rationally designing stimuli-responsive hydrogels to be used for ultrasound-triggered drug delivery requires a thorough understanding of the parameters that affect hydrogel response (Figure 3). These key factors are: bond strength, molecular weight, degree of polymerization, chain units, polymer structure, shape, and molecular assembly [33,75,76]. Rationally designing hydrogels to be as sensitive to ultrasound as possible is critical, as it will greatly decrease the chances of adverse biological effects [12,58].

These parameters are crucial when rationally designing stimuli-responsive hydrogels. Drug release from polymer systems requires relatively low amounts of energy to break, when paired with weaker bonds [77,78,79]. Molecular weight distribution also affects the responsiveness and location of mechanical force acting along a polymer chain [80,81,82]. The degree of polymerization and chain units influence the mechanochemical activity of polymeric materials [83,84,85]. Polymer structure and shape both play a role in the sonomechanical effects of ultrasound on materials [86,87,88]. The designed molecular assembly can also influence the mechanochemical activity of the materials [89,90,91]. The amount of energy used will be lowered by implementing these factors into the design of hydrogel matrices, which will also decrease the chances of surrounding tissue damage.

The factors involving the structure of a stimuli-responsive hydrogel have large effects on drug delivery, but another important parameter is the embedded payload or carrier within the hydrogel matrix. Possible embedded nanocarriers include microbubbles [92], nanoparticles [93,94,95], liposomes [92], loaded nanodroplets [72,96], and micelles [97,98]. Cells can be placed into hydrogel matrices for direct diffusion into the surrounding area [65] or aided by nanocarriers for increased targeting specificity [72]. Proteins have been diffused from hydrogels without direct targeting [99,100,101], or aided by nanocarriers in drug delivery systems [102]. Payloads such as drugs can also be directly diffused from hydrogels [103], or aided by nanocarriers for targeted drug delivery [104]. The rational design of hydrogels for ultrasound-triggered drug release is dependent on both the structural factors of the matrix and the embedded materials within the hydrogel.

While hydrogel matrices affect the response to focused ultrasound, the specific parameters of the applied ultrasound also influence the outcome. Two types of ultrasound can be used, either High-Intensity Focused Ultrasound (HIFU) or Low-Intensity Focused Ultrasound (LIFU), each being beneficial for different applications [37,66,69]. LIFU is advantageous for applications involving reversible cellular effects [15] and increased tissue regeneration [105]. For instance, Kearney et al. [93] and Levingstone et al. [106] used LIFU at 2.5 min per hour for 5 h with an intensity of 9.6 mW/cm^2^ to induce bone regeneration aided by BMP-2 release. For applications involving irreversible cell death or tissue ablation, HIFU would most likely be preferred [107]. For example, HIFU was used by Meng et al. [108] and Zhu et al. [109] at a 50% duty cycle with intensities of 6 W/cm^2^ and 1 W/cm^2^, respectively, to promote release and uptake in tumor systems.

Ultrasound has proven to be both safe and ethical for in vivo use in a variety of applications [15,16]. The Food and Drug Administration (FDA) has defined safety guidelines for ultrasound exposure [15]. Criteria such as the mechanical index, thermal index, spatial peak pulse average intensity, and spatial peak temporal average intensity have been defined to stipulate the maximum allowed ultrasound exposure [58,110,111]. Adverse biological effects can be avoided during in vivo ultrasound studies when following these.

Drug delivery applications must be fully understood to rationally design hydrogels specific for each application. The two main applications for ultrasound drug delivery via hydrogel systems are tissue engineering and cancer therapy. Each application features a variety of hydrogel systems, ultrasound parameters, delivery methods, and drugs used.

## 5. Tissue Engineering Applications

**Figure 4:**
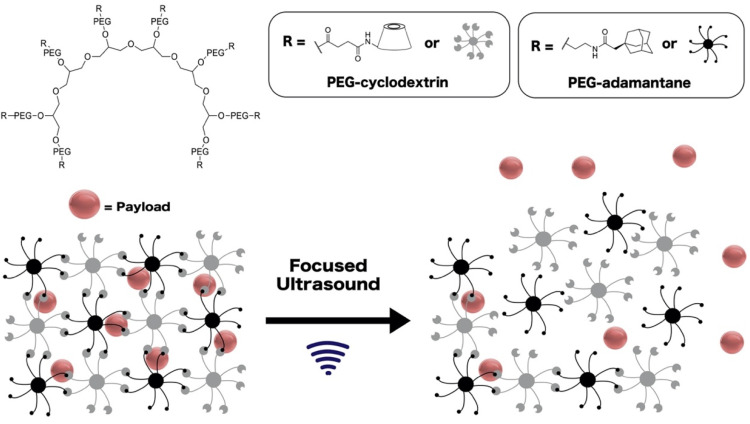
Design of ultrasound-responsive supramolecular PEG hydrogels crosslinked with a host-guest interaction between PEG-β-cyclodextrin and PEG-adamantane. This hydrogel matrix was developed by Yamaguchi et al. [100] and used for the controlled delivery of protein payloads.

Ultrasound has traditionally been used for imaging tissue and bone defects, but is more recently being used to control drug release from responsive hydrogel systems with spatiotemporal control. Injectable hydrogels have been chosen as drug release systems due to their high capabilities of drug loading and biocompatibility [112,113]. More advantages of the hydrogel networks come from their ability to act as scaffolds and carry therapeutic materials for release [113,114]. For instance, Yamaguchi and al. [100] developed supramolecular PEG hydrogels crosslinked with a host-guest interaction between PEG-β-cyclodextrin and PEG-adamantane. Embedded protein payloads were released in a site-specific manner from these hydrogels during exposure to focused ultrasound (Figure 4).

Tissue engineering hydrogel systems are rationally designed for a specific use, meaning each system has its own application, polymeric backbone, and delivery method (Table 1). Some of the tissue engineering applications include bone regeneration [65,93,106], cartilage repair [72,115], and skin repair [116]. The polymeric backbone of responsive hydrogels includes materials such as alginate [93,106], chitosan [72,115], cellulose [116], fibrin [117,118,119], and collagen [65]. The loading of these systems is made up of cells, proteins, or drugs. These ultrasound-responsive hydrogels then release their embedded payloads when exposed to focused ultrasound. This release occurs both with and without nanocarriers to aid in targeting. Ultrasound also proved to be safe when used in vivo for the delivery of angiogenic growth factors [117,119,120].

## 6. Applications for Cancer Therapy

**Figure 5:**
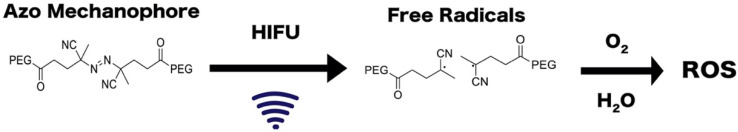
Design of ultrasound-responsive mechanophores embedded into PEG hydrogels, generating reactive oxygen species (ROS) when activated by high-intensity focused ultrasound. This hydrogel matrix was developed by Kim et al. [134] and used for the selective elimination of cancer cells in vitro.

Ultrasound has successfully been used as a minimally invasive diagnostic tool for the detection and follow-up of cancer patients [26,107,121] and for analyte detection [122,123,124,125]. Ultrasound has also been used for cancer treatment due to its effective real-time capabilities in imaging and has more recently been used for drug delivery from responsive hydrogel systems [126,127,128,129,130,131]. Like the hydrogel systems used for drug delivery in tissue engineering, these hydrogels were chosen due to their high loading efficiencies, stability, and flexibility [17]. The hydrogel systems could be loaded with either therapeutic drugs [132] or contrast agents for cancer [133]. For instance, Kim and al. [134] embedded mechanophores into PEG hydrogels. When activated by ultrasound, the mechanophores generated free radicals that converted to free oxygen species effectively killing melanoma and breast cancer cells in vitro (Figure 5).

The hydrogel systems that are rationally designed for cancer therapeutics cover a wide variety of applications, polymer systems, materials delivered, and nanocarriers used. Some of these applications include breast cancer [134,135,136], melanoma [103,134], tumor systems [108,109,137], and general cancer therapy [104]. Hydrogel polymer systems include alginate [103,135], PEG [134], OEGMA [108], hyaluronic acid [104], polylysine [136], chitosan [109], and silk fibroin [137]. These systems are used to deliver a variety of drugs, proteins, cells, and therapeutics payloads (Table 2). This delivery is completed both with and without the aid of nanocarriers within the system to complete the task of drug delivery. Ultrasound also proved to be safe when used in vivo for the delivery of antitumor agents such as doxorubicin or mitoxantrone [99,108,135].

## 7. Conclusions

Ultrasound-responsive hydrogels have been developed using a wide range of methods for delivery applications ranging from cancer therapeutics to bone regeneration. Ultrasound offers great advantages as an external trigger. It is localized, non-invasive, has deep tissue penetration, and offers real-time feedback by sonography. Ultrasound can also be focused to a small region of space and transfer acoustic energy via different thermal or non-thermal mechanisms.

We envision that future hydrogel delivery platforms will be custom-tailored for the chosen embedded payload in order to create synergistic effects between the payload and the ultrasound application in a specific tissue. One promising area of development is the use of thermoresponsive Diels-Alder linkers to crosslink polymeric hydrogels. This Click Chemistry reaction presents several advantages, it can be conducted in aqueous solution, it is highly efficient, it does not require a catalyst, and it is thermally reversible. When triggered by heat, the retro Diels-Alder reaction yields the original reactants. The chemical composition of these linkers can be modified to adjust the forward and reverse energy barriers, allowing to fine-tune the associated payload release kinetics.

Drug delivery systems could also be designed to respond to a combination of external focused ultrasound and internal physiological trigger (pH, enzyme, redox, or temperature) to combine their benefits. Upcoming platforms might also try to leverage the ability of ultrasound to facilitate transdermal delivery and enhanced uptake in cells and tissues.

Focused ultrasound will continue to be used in all types of drug delivery applications due to its ability to deliver payloads on-demand with spatiotemporal control. The interactions between acoustic mechanisms and drug delivery mechanisms will be critical in defining a specific application for an ultrasound-responsive hydrogel delivery system. Acoustic energy can be transmitted either via thermal mechanisms or non-thermal mechanisms. Delivery platforms for cancer therapy will most likely be dependent on high-intensity focused ultrasound due to its ability to invoke a thermal mechanism in solid tumors. Low-intensity focused ultrasound will be used for tissue engineering thanks to its capability to enhance uptake in cells and tissue.

The main challenges for future ultrasound-responsive drug delivery systems are related to the safety of the focused ultrasound, especially for high-intensity focused ultrasound. Running a system with the lowest amount of energy required is always beneficial to mitigate any potential damage. Future ultrasound-responsive hydrogels will most likely be rationally designed to reduce the amount of energy required to trigger the release and minimize any risk of damage to the surrounding tissues. Guidance from regulating agencies such as the safe operating guidelines developed by the FDA will be helpful in the future to safely translate to the clinic the emerging early-stage strategies currently explored in vitro.

Overall, ultrasound has a tremendous potential to become increasingly popular as a stimulus for on-demand drug delivery platforms and to improve the clinical outcome of a variety of advanced drug delivery applications.

## Figures and Tables

**Table 1:** Characteristics of ultrasound-responsive hydrogels for tissue engineering applications.

Application	Hydrogel Polymer System	Payload	Ultrasound Parameters	Reference
Bone Regeneration	Alginate Hydrogel	BMP-2	2.5 min/h for 5 h Amplitude of 25% 9.6 mW/cm^2^	[106]
Cartilage Repair	Chitosan Hydrogel	BMSCs aided by nanocarriers	1 MHz 2–3 W/cm^2^ 20–30% duty cycle	[72]
Bone Regeneration	Alginate Hydrogel	BMP-2 conjugated gold nanoparticles	2.5 min/h for 5 h Amplitude of 25% 9.6 mW/cm^2^	[93]
Cartilage Repair	Chitosan Hydrogel	Kartogenin on microparticles	2 and 5 min intervals	[115]
Skin Repair	Cellulose Hydrogel Film	Mimosa drug	LIFU 23, 43, and 96 kHz 5–30 W	[116]
Vascularization	Fibrin Hydrogel	bFGF release	100 Hz, 6.1 MPa 5.4 μs pulse	[117]
Bone Regeneration	Collagen Hydrogel	Osteoblasts	LIPUS 1 MHz, 1 kHz, 1 Hz Duty cycle: 20%, 50% or 100% 30 and 150 mW/cm^2^	[65]

**Table 2:** Characteristics of hydrogel polymer systems used for cancer therapy.

Application	Hydrogel Polymer System	Payload	Ultrasound Parameters	Reference
Breast Cancer Treatment	Alginate Hydrogel	Mitoxantrone	HIFU 9.6 mW/cm^2^ 5 min pulses/h, /2 h, or /24 h	[135]
Melanoma and Breast Cancer	PEG Hydrogel	AZO-Mechanophores for MDT	HIFU 550 kHz 115 W/cm^2^, 1.9 MPa 10 s on and 20 s off	[134]
Tumor Systems	Nanocomposite Hydrogel	Nanovaccines (ORP nanoparticles)	HIFU 40 kHz 6 W/cm^2^, 50% duty cycle	[108]
Cancer Therapy	Hyaluronic Acid Hydrogel	Doxorubicin loaded gold nanoparticles	HIFU 10, 20, 30, or 50 W 30 or 60 min 1.5 MHz 50% Duty cycle 1 Hz pulse frequency	[104]
Melanoma	Alginate Hydrogel	Mitoxantrone	HIFU 20% or 40% amplitude 1 or 5 min	[103]
Breast Cancer	Polylysine Nanogel	Epirubicin aided by ICAM-1	HIFU 15 or 30 min 10 W	[136]
Tumor Systems	Chitosan Hydrogel	Piezoelectric Tetragonal BaTiO_3_	HIFU 1 MHz, 1 W/cm^2^ 50% duty cycle 1, 2, 3, 4, 5, or 10 min	[109]
Tumor Systems	Silk Fibroin Hydrogel	Vincristine	HIFU 1, 2, or 3 W 14.3, 28.5, or 42.8 W/cm^2^ 20 s or 1 min	[137]
